# *Sinorhizobium meliloti* 1021 Exopolysaccharide as a Flocculant Improving Chromium(III) Oxide Removal from Aqueous Solutions

**DOI:** 10.1007/s11270-014-2052-4

**Published:** 2014-07-05

**Authors:** Katarzyna Szewczuk-Karpisz, Małgorzata Wiśniewska, Małgorzata Pac, Adam Choma, Iwona Komaniecka

**Affiliations:** 1 Department of Radiochemistry and Colloid Chemistry, Faculty of Chemistry, Maria Curie Sklodowska University, M. Curie Sklodowska Sq. 3, 20-031 Lublin, Poland; 2 Department of Genetics and Microbiology, Faculty of Biology and Biotechnology, Maria Curie Sklodowska University, Akademicka 19 Street, 20-033 Lublin, Poland

**Keywords:** *Sinorhizobium meliloti* 1021 exopolysaccharide, Chromium(III) oxide, Adsorption, Zeta potential, Potentiometric titration, Suspension destabilization

## Abstract

Chromium(III) oxide is an amphoteric, dark green solid. This most stable dye is widely used in construction and ceramic industries as well as in painting. In this study, the attempt is made to determine flocculating properties of exopolysaccharide (EPS) synthesized by the bacteria *Sinorhizobium meliloti* 1021, which would increase the efficiency of chromium(III) oxide removal from sewages and wastewaters. The conditions under which EPS is the most effective destabilizing component of chromium(III) oxide suspension have been determined too. In order to characterize the structure of electric double layer formed at the solid/supporting electrolyte (EPS) solution interface, electrokinetic potential measurements and potentiometric titration were performed. The EPS amount adsorbed on the chromium(III) oxide surface as a solution pH function was also measured. Moreover, the stability of Cr_2_O_3_ suspension in the absence and presence of *S. meliloti* 1021 EPS was estimated. The pooled analysis of all obtained results showed that EPS causes chromium(III) oxide suspension destabilization in the whole examined pH range. The largest change in the system stability before and after the polymer addition was observed at pH 9. It is probable that under these conditions bridging flocculation occurs in the examined system.

## Introduction

Drinking water shortage in many world regions forces searching for new flocculants and destabilizing factors of possibly high efficiency in solid particles removal from sewages and wastewaters. Microbial flocculants (MBF) create a great opportunity for improving the efficiency of wastewater and sewage treatment. The macromolecular substances like glycoproteins, polysaccharides and nucleic acids are produced by microorganisms during their growth. MBFs accelerate solid particles aggregation and make flocs size greater (Zhang et al. 2009; Lian et al. 2008). Compared to the traditional synthetic flocculants, MBFs are biodegradable, non toxic and their use does not cause secondary water pollution (Yokoi et al. 1995). The MBF mechanism may be based on adsorbent surface charge neutralization by polymer macromolecules of opposite charge or bridging flocculation during which one polymer chain adsorbs on at least two solid particles surface (Tong et al. 1999). In recent years flocculating properties of many bacterial substances, inter alia, bioflocculants produced by *Klebsiella* sp. TG-1 (Liu et al. 2013), *Pleurochrysis certerae* (Lee et al. 2009), *Bacillus* spp. UPMB13 (Zulkeflee et al. 2012) and *Bradybacterium* sp. (Nwado et al. 2013) have been described.

**Fig. 1:**
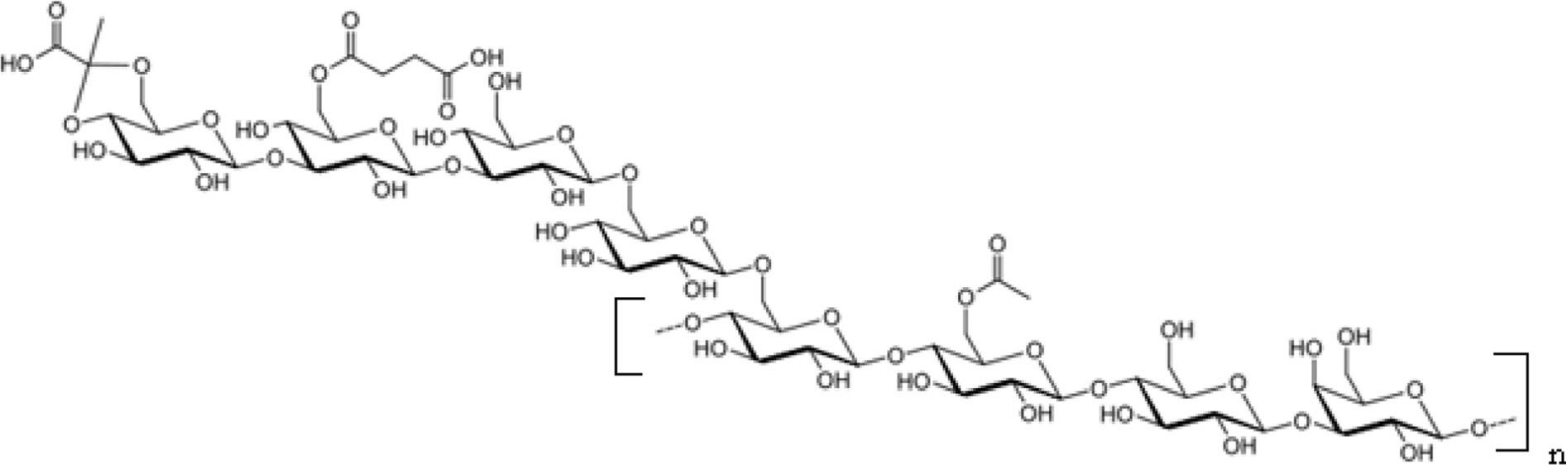
Structure of the *Sinorhizobium meliloti* 1021 exopolysaccharide monomer

Exopolysaccharides (EPS) are macromolecular compounds synthesized in large quantities by all soil bacteria belonging to the *Rhizobiaceae* family. They play a key role in the establishment of symbiosis between bacteria and legume plants (Janczarek et al. 1999). In this paper there is used EPS synthesized by *Sinorhizobium meliloti* 1021. These bacteria can produce two types of EPS, defined as EPS I and EPS II. The first type (EPS I) is succinoglycan, composed of octasaccharide subunits containing seven glucose and one galactose molecules joined by β-1, 3, β-1, 4 and β-1, 6 glycosidic bonds (Skorupska et al. 2006). Succinoglycan has a polysaccharide skeleton modified by acetyl, pyruvyl and succinyl substituents (Reinhold et al. 1994). The structure of octasaccharide *S. meliloti* 1021 EPS unit is shown in Fig. 1 (Simsek et al. 2007). EPS II is galactoglucan composed of disaccharide units containing acetylated glucose and galactose substituted by the puryvic acid residue linked by β-1, 3 and α-1, 3 glycosidic bonds. This EPS is synthesized either in the phosphorus compounds poor medium or due to mutation of specific regulatory genes (Bahlawane et al. 2008; Pellock et al. 2002). Both EPS types are produced in two fractions: high molecular weight (HMW) and low molecular weight (LMW). The HMW fraction includes EPS macromolecules made up of several hundred/a few thousand subunits. Their weight is in the range 10^6^–10^7^ Da (Gharzouli et al. 2013). The LMW fraction is composed of monomers, dimers and trimers in the case of EPS I or oligomers containing 15–20 units in the case of EPS II (Wang et al. 1999).

Chromium(III) oxide is a finely crystalline solid as well as the most stable green dye (Gattens and Stout 1966). Due to its extensive use in many industries, the Cr_2_O_3_ presence in wastewaters is inevitable. Getting of poorly treated wastewaters containing chromium(III) oxide is highly harmful for aquatic organisms. Colourful substances inhibit the penetration of solar radiation into natural water deep layers and thus reduce the photosynthesis efficiency (Świderska-Bróż 1993). Moreover, Cr_2_O_3_ makes water turbid which causes that it can not be consumed by people. Removal of different chromium forms from sewages and wastewaters has been the subject of numerous scientific publications (Gupta and Rastogi 2009; Gupta et al. 2011). For example, the procedure of activated carbon production from tires (Gupta et al. 2013) and fertilizer plant wastes (Gupta et al. 2010) has been described. In previous years, our team has determined the poly(acrylic acid) flocculating ability for the chromium(III) oxide suspension (Wiśniewska and Szewczuk-Karpisz 2013).

This aim of this study was to assess *S. meliloti* 1021 EPS as a substance accelerating chromium(III) oxide removal from sewages and wastewaters. Experimental works (i.e., stability, EPS adsorption level, electrokinetic potential measurements and potentiometric titration) provided the results enabling determination of conditions under which EPS is the most efficient factor of Cr_2_O_3_ suspension destabilization. These results can promote development of innovative sewage and wastewater treatment procedures.

## Experimental

### Materials

#### Adsorbent

Chromium(III) oxide (*POCh*) with a surface area of 7.12 m^2^/g (BET method) was used as the adsorbent. Prior to the experiments metal oxide was washed to remove inorganic ions using doubly distilled water to achieve the supernatant conductivity of less than 3 mS/cm. Then the adsorbent was dried and crushed in a porcelain crucible.

#### Exopolysaccharide

**Fig. 2:**
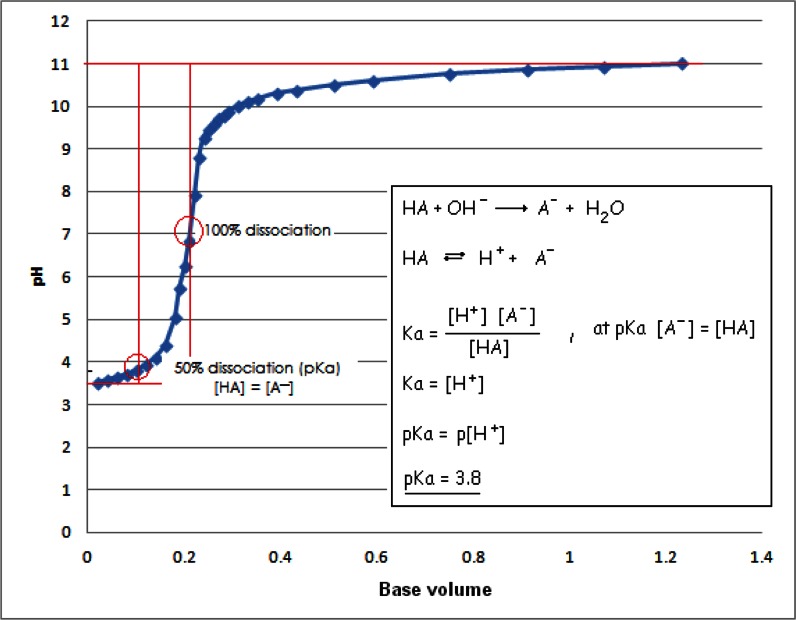
Determination of the p*K*_a_ value of *Sinorhizobium meliloti* 1021 exopolysaccharide. Dependence of solution pH value on the added base volume

The p*K*_a_ value of EPS synthesized by *S. meliloti* 1021 was determined by the graphical method using the dependence of solution pH on the added base volume. This dependence was obtained using potentiometric titration of EPS solution (Fig. 2). It was found that at pH 3.8 the number of dissociated and undissociated carboxylic groups in EPS macromolecules is identical. By using the following formulas (Minczewski and Marczenko 1965):

1 $$ \mathrm{pH}-\mathrm{p}{K}_{\mathrm{a}}=\frac{\left[ RCO{O}^{-}\right]}{\left[\mathrm{RCOOH}\right]} $$

2 $$ \mathrm{pH}-\mathrm{p}{K}_{\mathrm{a}}= \log \frac{\alpha}{1-\alpha}, $$

the dissociation degrees (*α*) and the ratio of the dissociated carboxylic groups concentration and the undissociated carboxylic groups concentration ([RCOOH]/[RCOO^−^]) of EPS as a function of solution pH were calculated. The obtained results are presented in Table 1. It was found that the higher solution pH, the greater EPS dissociation degree. At pH 7.6 and 9, almost all carboxylic groups in the bacterial polysaccharide macromolecules are dissociated.

**Table 1:** Ratio of COOH groups concentration to COO^−^ groups concentration in EPS molecule and their dissociation degree (α) as a function of solution pH value

pH	[COOH]/[COO^−^]	*α*
3	6.3	0.136
3.8	1	0.5
4.6	0.158	0.863
7.6	0.000159	0.9998
9	0.0000063	0.99999

### Methods

#### EPS Isolation

*S. meliloti* 1021 was grown in 1 l of the liquid 79CA medium supplemented with succinate (Vincent 1970). The culture was grown for 48 h under continuous aeration by shaking (120 rpm) at 28 °C. The bacteria were removed from the medium and cooled by centrifugation (30 min, 9 000 rpm). The supernatant was concentrated using a vacuum evaporator and then the obtained concentrated liquid was dialyzed for 3 days by distilled water. EPS was precipitated from the concentrated liquid by adding 3 volumes of cold ethanol. EPS precipitate was resuspended in water and lyophilized.

#### Stability Measurements

Stability measurements of chromium(III) oxide suspension in the presence and absence of *S. meliloti* 1021 EPS were made using a turbidimeter *Turbiscan Lab* ^*Expert*^ with a cooling module *TLAb Cooler*. The suspensions were prepared by adding 0.02 g of chromium(III) oxide to 20 ml of supporting electrolyte solution (0.01 M NaCl). The suspensions containing EPS were prepared by adding an identical Cr_2_O_3_ portion to 0.01 M NaCl solution. Each suspension was sonicated for 3 min. After EPS addition (1 ppm) and suspension pH determination (3, 4.6, 7.6 or 9 ± 0.05) the stability measurement started.

During the measurement the light beam with the wavelength of 880 nm passed through the sample. The turbidimeter is equipped with two detectors: (1) the transmission detector registering the light passing through the sample, (2) the backscatter detector registering the light scattered at an angle of 135°. The results obtained in the form of curves of transmission and backscattering light passing through the sample. A single measurement lasted 15 h, during which the transmission and backscatter curves were recorded every 15 min. All measurements were performed at 25 °C. In addition, computer software working with the turbidimeter allowed calculation of Turbiscan Stability Index (TSI) and the average size of aggregates (flocs) formed in the examined systems. The TSI factor was determined from the following formula:

3 $$ \mathrm{TSI}=\sqrt{\frac{{\displaystyle {\sum}_{i=1}^n{\left({x}_i-{x}_{BS}\right)}^2}}{n-1}}, $$

where *x*_*i*_ is the average backscatter for each minute of measurement, *x*_BS_ is the average *x*_*i*_ value, and *n* is the scans amount.

#### Adsorption Measurements

To determine the concentration of *S. meliloti* 1021 EPS, the method developed by Dubois et al. (1956) was used. In brief, 5 ml of 95 % sulfuric acid and 50 μl of 80 % phenol were added to 2 ml of the polysaccharide solution at the same time. The intensity of the obtained colour was measured spectrophotometrically at a wavelength of 490 nm using a UV–Vis spectrophotometer Carry 100 (*Agilent Technologies*). The standard curve was prepared by measuring the absorbance of solution series with the EPS concentrations equal to 10, 30, 50, 70, 100, 150, 200 ppm.

Adsorption amount was determined by the difference in EPS concentration before and after the adsorption process. After preparing 10-ml solutions containing 0.01 M supporting electrolyte (NaCl) and the appropriate EPS amount that gave its final concentration equal to 10, 30, 50, 70, 100, 150 or 200 ppm, 0.05 g chromium(III) oxide was added to each of them. Solution pH was determined to be 3, 4.6, 7.6 or 9 using 0.1 M HCl and 0.1 M NaOH. Adsorption was carried out under continuous shaking (about 120 rpm) at 25 °C for 20 h (until the system equilibrium was reached). The adsorption time was determined on the basis of kinetic measurements, while the metal oxide sample weight was determined after taking into account its surface area. After adsorption completion suspensions were centrifuged (10 min, 4 000 rpm) and then polysaccharide concentration was determined in the supernatant. One result was the average of three measurements. The measurement error did not exceed 5 %.

#### Electrokinetic Measurements

##### Potentiometric Titration

In the potentiometric titration method the surface charge density (*σ* ^0^) is determined based on the difference in base volume added to a suspension containing a polymer and a supporting electrolyte solution in order to achieve a specific pH value using the formula (Janusz et al. 1997):

4 $$ {\sigma}^0=\frac{\varDelta V\cdot c\cdot F}{m\cdot {S}_{\mathrm{w}}}, $$

where Δ*V* is the difference in base volume added to a suspension and a supporting electrolyte solution that lead to specific pH value (*ΔV* = *V*_s_ − *V*_e_), *c* is the base concentration, *F* is the Faraday constant, *m* is the metal oxide mass in a suspension, and *S*_w_ is the metal oxide surface area.

Potentiometric titration was conducted under the thermostatic conditions (25 °C) using the 'titr_v3' computer program developed by W. Janusz and the apparatus consisting of: thermostat RE 204 (*Lauda*), glass and calomel electrode (*Backman Instruments*), pHmeter PHM 240 (*Radiometer*), automatic microburette Dosimat 765 (*Metrohm*) connected with the computer.

At first the supporting electrolyte (0.01 M NaCl) solution and the *S. meliloti* 1021 EPS solution with the concentration of 1 ppm were titrated. Next, the titrations of chromium(III) oxide suspension in the presence and absence of the bacterial polymer were conducted. To prepare suspensions 1.5 g metal oxide was used. EPS concentration in the examined suspensions was equal to 0.1, 1, 10 or 50 ppm. All systems were titrated with the 0.1 M NaOH solution.

##### Zeta Potential Measurements

Electrokinetic potential measurement started with the preparation of 500 ml chromium(III) oxide suspension by adding 0.03 g of solid to the solution containing 0.01 M supporting electrolyte (NaCl) and appropriate EPS amount giving its final concentration of 0.1, 1, 10 or 50 ppm. After 3-min sonication suspensions were poured into five Erlenmeyer flasks and their pH values were adjusted to 3, 4.6, 6, 7.6 and 9 ± 0.05. Then the zeta potential was measured using Zetasizer 3000 (*Malvern Instruments*); the apparatus was washed twice before each measurement. One result was the average of five measurements. The measurement error did not exceed 5 %.

In the same way, the zeta potential of chromium(III) oxide particles in the supporting electrolyte solution (0.01 M NaCl) without the bacterial polymer was measured.

## Results and Discussion

### Stability of Chromium(III) Oxide Suspension in the Absence and Presence of *S. meliloti* 1021 Exopolysaccharide

**Fig. 3:**
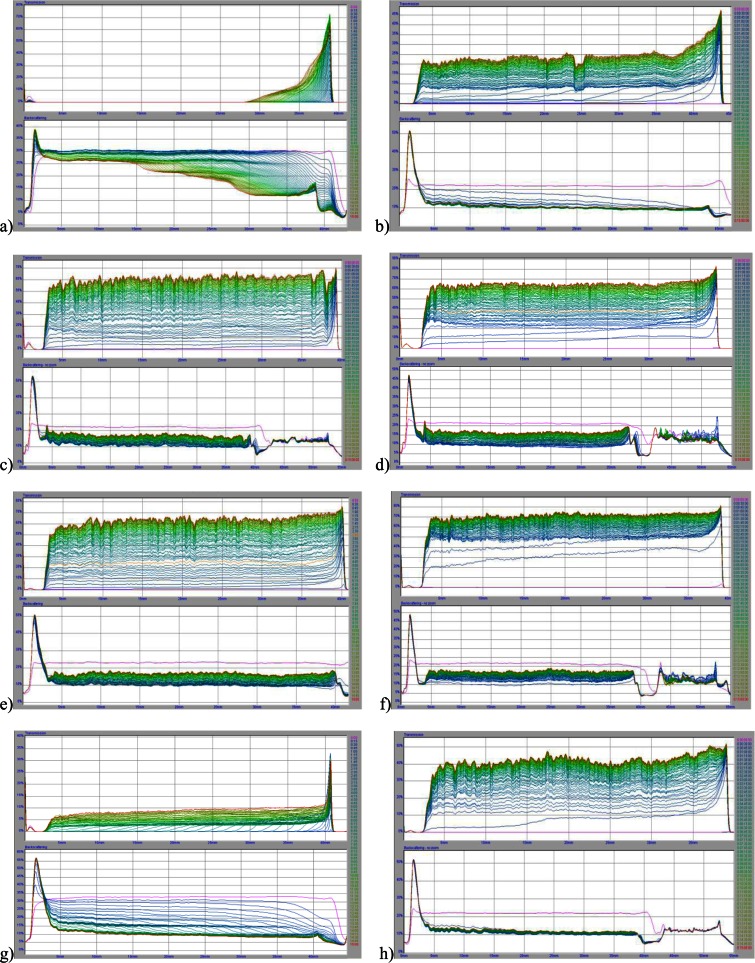
Transmission and backscatter curves for the systems: **a** Cr_2_O_3_ at pH 3, **b** Cr_2_O_3_–EPS at pH 3, **c** Cr_2_O_3_ at pH 4.6, **d** Cr_2_O_3_–EPS at pH 4.6, **e** Cr_2_O_3_ at pH 7.6, **f** Cr_2_O_3_–EPS at pH 7.6, **g** Cr_2_O_3_ at pH 9, **h** Cr_2_O_3_–EPS at pH 9. *C*_EPS_ = 1 ppm, *C*_NaCl_ = 0.01 M

The chromium(III) oxide suspension stability in the absence and presence of *S. meliloti* 1021 EPS was measured using the turbidimeter. The results were obtained in the form of light transmission and backscattering curves. Generally, the high level of backscatter and low transmission indicate a high suspension stability. On the other hand, light backscatter decrease and transmission increase provide lower system stability. The analysis of mutual curve arrangement allows determination of dynamics of the processes occurring in the sample during the measurement. A large distance between the curves is equivalent to the lower system stability, and thus the rapid particles sedimentation. In turn, the curve overlap indicates greater suspension stability and lower rate of particle sedimentation. The measurement results are shown in Fig. 3.

In addition, computer software working with the turbidimeter makes calculation of the TSI as well as the average size of aggregates (flocs) formed in the examined suspensions possible. The TSI coefficient is a parameter used in systems stability assessment. The higher the TSI value, the lower the suspension stability. The TSI values obtained for the chromium(III) oxide suspension with and without the bacterial polysaccharide as a function of solution pH are presented in Table 2. The average size of aggregates (flocs) formed in the systems under examination is shown in Table 3.

**Table 2:** The Turbiscan Stability Index (TSI) values for chromium(III) oxide suspension in the absence and presence of EPS (1 ppm) calculated on the basis of data obtained at 15 h using Eq. 3

System	Turbiscan Stability Index			
	pH = 3	pH = 4.6	pH = 7.6	pH = 9
Cr_2_O_3_	12.76	33.28	62.91	29.28
EPS–Cr_2_O_3_	25.59	37.9	72.5	61.85

**Table 3:** The average size of aggregates (flocs) in the suspension of chromium(III) oxide in the absence and presence of EPS (1 ppm) at 15 h

System	Average size of aggregates (flocs) (μm)			
	pH = 3	pH = 4.6	pH = 7.6	pH = 9
Cr_2_O_3_	0.077	0.506	0.529	0.127
EPS–Cr_2_O_3_	0.0852	0.568	0.559	0.625

The analysis of the light transmission and backscatter graphs and the stability factor (TSI) values showed that the chromium(III) oxide suspension without *S. meliloti* 1021 EPS has the lowest stability at pH 7.6 (TSI = 62.91). What is more, the most stable is the Cr_2_O_3_ suspension at pH 3 (TSI = 12.76). At pH 4.6 and 9 the intermediate TSI results were found to be 33.28 and 29.28, respectively. Moreover, it was stated that in less stable systems larger aggregates are formed. The particle clusters of the largest diameter (0.529 μm) were observed in the suspension at pH 7.6, which had the lowest stability of all studied systems without polysaccharide. In turn, aggregates of the smallest sizes (0.077 μm) were present at pH 3 — the most stable system of all chromium(III) oxide suspensions without EPS.

Regardless of suspension pH value the bacterial polysaccharide addition results in the system stability decrease and the growth of TSI. The greatest stability coefficient value was found in the suspension at pH 7.6 (TSI = 72.5). But, the most pronounced decrease in the system stability is at pH 9. Under these conditions the polysaccharide addition caused the increase in the TSI values from 29.28 to 61.85, i.e., about 30 units. It is worth noting that in the suspensions containing *S. meliloti* 1021 EPS, the formed flocs were larger than in the systems without the polymer. For example, at pH 4.6 polysaccharide presence changes the solid clusters size diameter from 0.506 to 0.568 μm. The biggest difference in the aggregate (floc) sizes in the suspension with and without the EPS was observed at pH 9 (Δ = 0.498 μm).

### Adsorption Amount of *S. meliloti* 1021 Exopolysaccharide on the Chromium(III) Oxide Surface

**Fig. 4:**
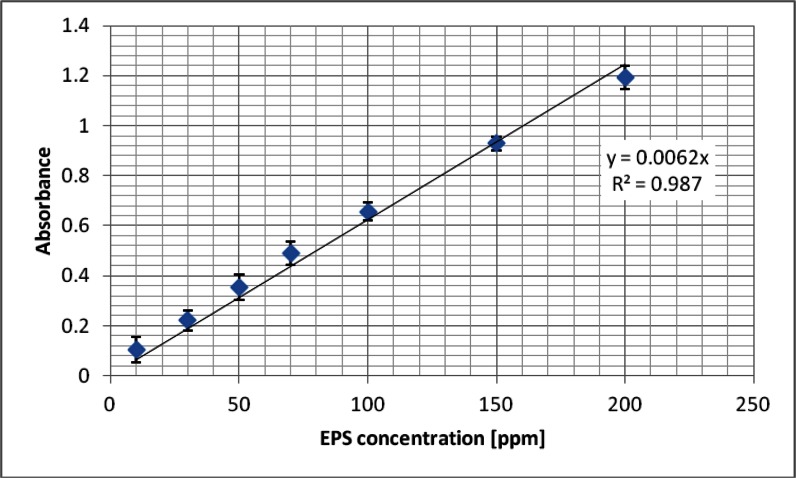
Calibration curve for *Sinorhizobium meliloti* 1021 exopolysaccharide

**Fig. 5:**
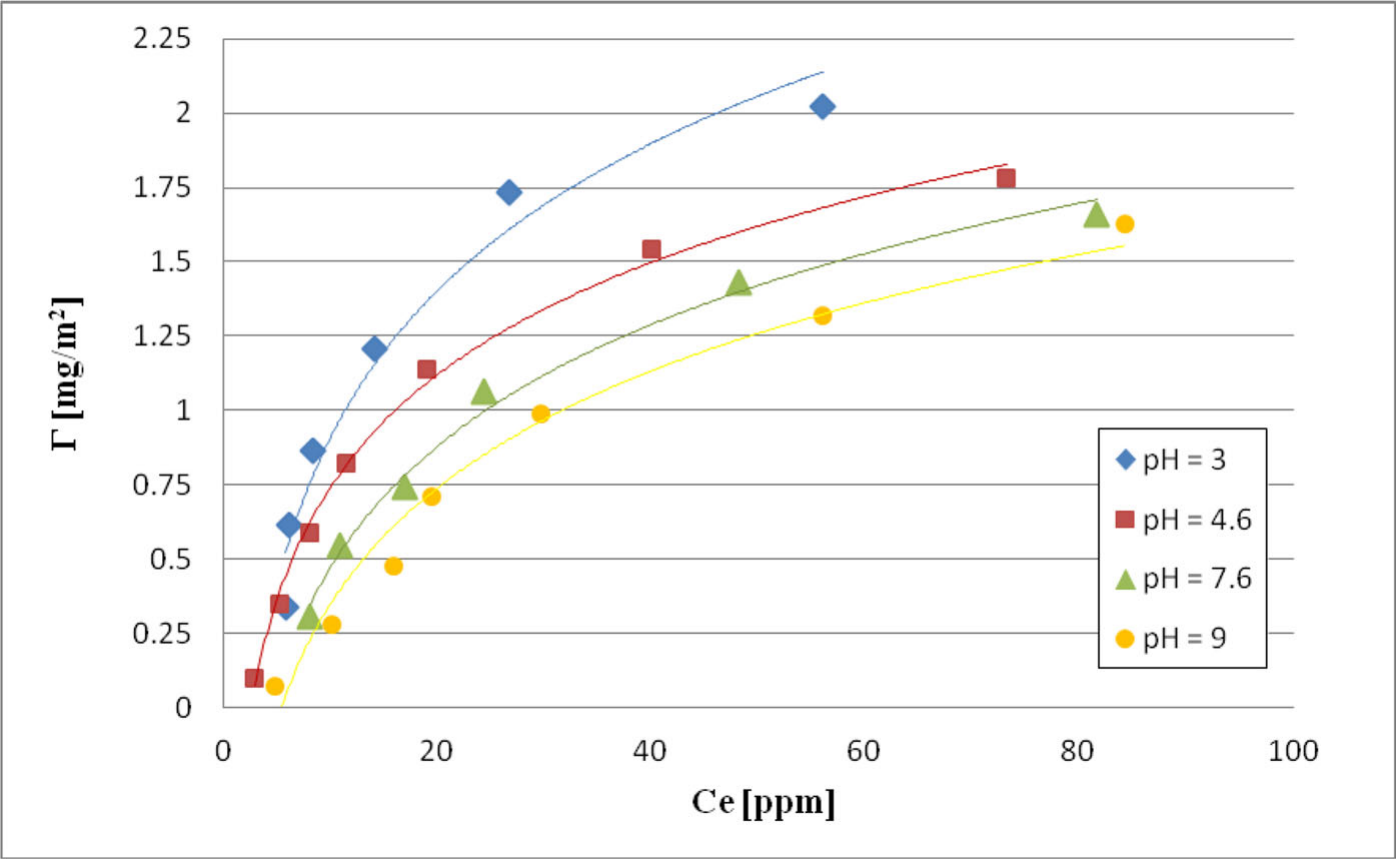
Adsorption isotherms of *Sinorhizobium meliloti* 1021 exopolysaccharide on the Cr_2_O_3_ surface at different pH

Knowledge of adsorption level and the dependence of EPS amount adsorbed on the chromium(III) oxide surface on the solution pH value is largely useful in explaining the observed changes in the suspension stability in the presence and absence of EPS. Adsorption was determined from the difference in the bacterial polysaccharide concentration in the solution before and after the adsorption process. Dependence of absorbance on the expolysaccharide concentration is shown in Fig. 4, while the obtained adsorption isotherms are shown in Fig. 5.

Analyzing the results it was found that the adsorption amount of *S. meliloti* 1021 EPS is largely dependent on solution pH. The largest amount of polymer adsorbed on the chromium(III) oxide particle surface was observed at pH 3. This is probably related to EPS macromolecule conformation and attractive electrostatic adsorbent–adsorbate interactions. Based on the results of the potentiometric titration it was calculated that the EPS p*K*_a_ value is about 3.8. This means that in solution of such pH in the EPS macromolecules the number of dissociated carboxylic groups is equal to that of the undissociated carboxylic groups. At pH <3.8 in the EPS macromolecules COOH groups prevail, while at pH >3.8 COO^−^ groups are dominant. At pH 3 the concentration ratio of COOH to COO^−^ groups is 6.3. Therefore, it can be assumed that the relatively small number of dissociated carboxylic groups in adsorbate macromolecules contributes to their coiled conformation. Such packed polymer structure allows adsorption of a greater number of EPS macromolecules on a unit adsorbent area. Moreover, the previously made potentiometric titration showed that the zero point of the chromium(III) oxide surface charge (pH_pzc_) is 7.6, which is equivalent that at pH 3 the adsorbent surface is positively charged. Thus, under these conditions, electrostatic attraction between the polymer macromolecules and the metal oxide particles occurs, which further contributes to the highest adsorption level at pH 3.

At pH 4.6 the amount of adsorbed bacterial polysaccharide is less than at pH 3. Under these conditions electrostatic adsorbent–adsorbate attraction is weaker due to the lower density of solid surface charge. Furthermore, at pH 4.6 EPS dissociation degree (*α*) is 0.86, which means that undissociated carboxylic groups number in the polysaccharide macromolecules is small. The advantage of COO^−^ groups in the polymer segments contributes to their mutual repulsion, which causes polysaccharide macromolecule expansion. Adsorption of these polymer chains is equivalent to the blocking of active sites on the adsorbent surface resulting in the decreasing of the EPS adsorption level.

At pH 7.6, the EPS adsorption amount is lower than at pH 4.6. Under these conditions the adsorbent surface has a zero charge (pH_pzc_) because the concentrations of positive and negative surface functional groups are equal. Therefore, there are no electrostatic interactions that could affect the adsorbed polymer amount. The observed adsorption level at pH 7.6 is primarily dependent on the EPS macromolecule structure. At such solution pH value, almost all carboxylic groups in the EPS macromolecules are dissociated (*α* = 0.99), which contributes to the occurrence of strong electrostatic repulsion between polymer segments. Macromolecules assume even more expanded conformation than it was at pH 4.6. This EPS structure causes that a single macromolecule takes even a larger part of the Cr_2_O_3_ surface during adsorption.

The minimum amount of bacterial polymer adsorbed on the metal oxide surface was found at pH 9. Under these conditions the EPS dissociation degree is equal to 0.99 and hence the macromolecule conformation at pH 9 is similar to that at pH 7.6. Moreover, at pH 9 the adsorbent surface is negatively charged which contributes to the electrostatic repulsion between the EPS macromolecules and the chromium(III) oxide particles. The above interaction is the main reason for reducing the adsorption level. It should be noted that under these adverse conditions a small amount of EPS is adsorbed on the Cr_2_O_3_ surface. It is evidence that in the system under examination the additional forces causing adsorption are present. They are so called hydrogen bonds which can form between the adsorbate carboxylic groups (both COOH and COO^−^) and all types of adsorbent surface groups (–CrOH, –CrOH_2_^+^,–CrO^−^). These linkages are present in the analyzed system in the whole investigated pH range, which also indicates the specific nature of adsorption.

As follows from the analysis the EPS adsorption amount on the Cr_2_O_3_ surface increases with the concentration growth. It is related to different degrees of *S. meliloti* 1021 EPS polymerization. Thus, EPS molecular weight changes in a specific range. In the suspensions containing a small amount of bacterial polysaccharide practically all EPS macromolecules are adsorbed on the solid surface. With the increasing EPS concentration longer chains start displacing the shorter ones on the adsorbent surface. Macromolecules of higher molecular weight are characterized by a higher affinity for the active sites on the Cr_2_O_3_ surface. This affinity is probably due to the higher values of adsorption free energy of the EPS chains characterized by higher mass.

### Surface Charge Density of Chromium(III) Oxide Particles in the Absence and Presence of *S. meliloti* 1021 Exopolysaccharide

**Fig. 6:**
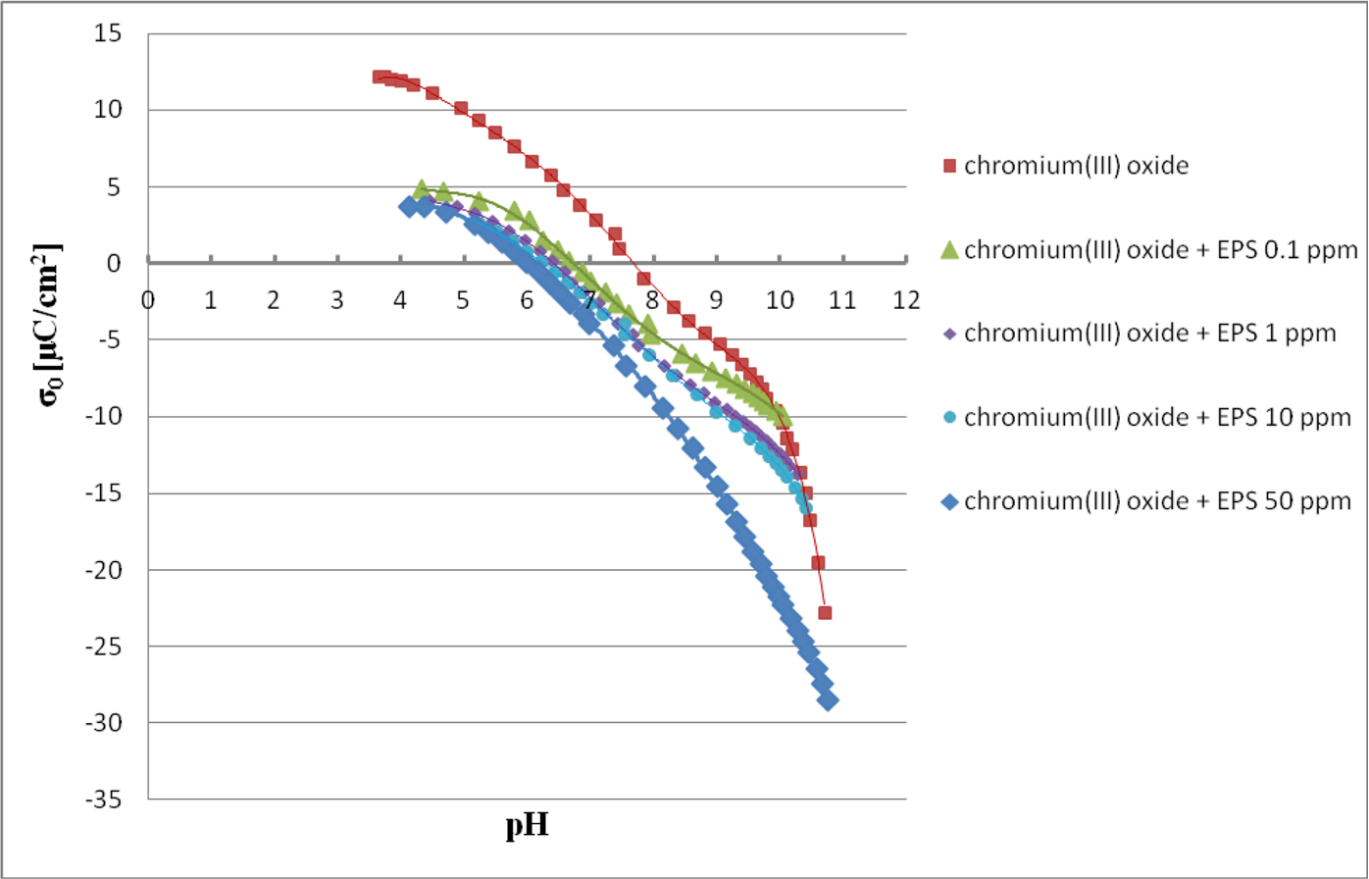
Surface charge density of chromium(III) oxide particles in the absence and presence of *Sinorhizobium meliloti* 1021 exopolysaccharide

Potentiometric titration allows to determine the sign and density of solid surface charge, which are important parameters characterizing the electrical double layer structure formed at the interface. In experiments chromium(III) oxide suspensions were titrated in the absence and presence of *S. meliloti* 1021 EPS. The results are shown in Fig. 6.

The obtained results showed that the chromium(III) oxide zero point charge (pH_pzc_) oxide is equal to 7.6. In the presence of the bacterial polymer this point is shifted to the acidic pH. When EPS with the concentration of 0.1 ppm is added to the suspension the pH_pzc_ value is approximately 6.7. The EPS presence with the concentration of 50 ppm causes the metal oxide pH_pzc_ change to approximately 5.9. Thus it can be concluded that the higher the bacterial polysaccharide concentration, the greater shift of the chromium(III) oxide pH_pzc_ to the acidic pH values.

It was observed that the *S. meliloti* 1021 EPS adsorption on the chromium(III) oxide surface contributes to the reduction in the adsorbent surface charge in the entire pH range. Moreover, the greater the reduction, the higher the solution pH. The pH value increase results in the increasing number of undissociated carboxylic groups in the polysaccharide macromolecules. These COO^−^ groups occurring in both solution layer indirectly adjoining to the solid surface and polymer segments not linked to the adsorbent (forming a loop and tail structures) are responsible for the metal oxide surface charge reduction. In addition, the above reduction becomes more pronounced with the increasing EPS concentration. It is associated with the previously mentioned displacement of macromolecules of lower molecular weight by macromolecules of higher molecular weight on the adsorbent surface. The longer polymer chains contain a greater number of functional dissociated groups than the shorter ones. During the adsorption EPS macromolecules of higher mass formed a layer involving more loops and tails rich in COO^−^ groups. The participation of these structures in the adsorption layer in the case of short EPS chains adsorption is relatively smaller, which is reflected in a smaller reduction in the adsorbent surface charge.

### Electrokinetic Potential of Chromium (III) Oxide Particles in the Absence and Presence of *S. meliloti* 1021 Exopolysaccharide

**Fig. 7:**
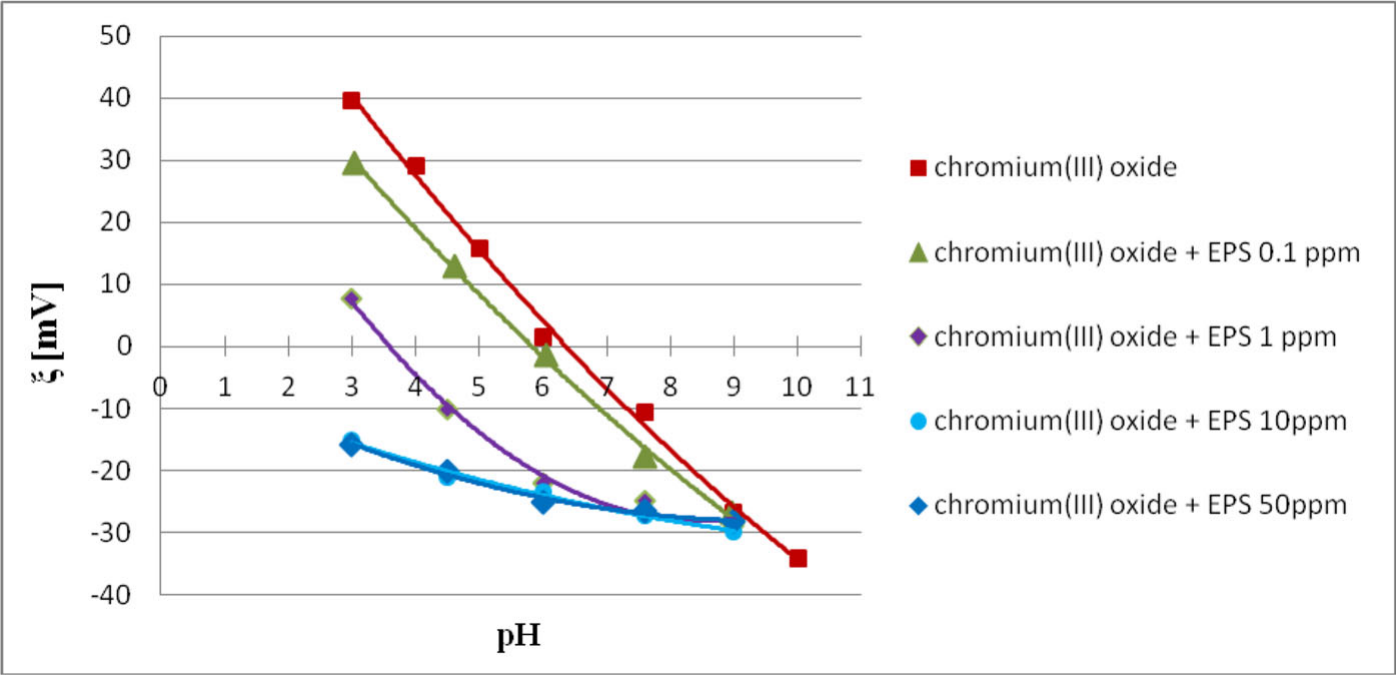
Zeta potential of Cr_2_O_3_ particles with and without *Sinorhizobium meliloti* 1021 exopolysaccharide

Knowledge of sign and density of adsorbent surface charge as well as chromium(III) oxide particles zeta potential (*ξ*) values allows a precise characterization of the electrical double layer structure formed at the chromium(III) oxide/supporting electrolyte (EPS) solution interface. In addition, the information about the electrokinetic potential values can be very helpful in explaining changes in the system stability as a result of polymer adsorption. The measured electrokinetic potential values are shown in Fig. 7.

It was found that the chromium(III) oxide isoelectric point (pH_iep_), i.e., the pH value at which the number of positive and negative groups on the slipping plane is identical, is about 6. The bacterial polysaccharide addition causes the pH_iep_ shift towards lower pH values. In the presence of EPS with the concentration of 0.1 ppm, the Cr_2_O_3_ isoelectric point value is about 5.75, and after the addition of 1 ppm EPS — about 3.5. High EPS concentrations (i.e., 10 and 50 ppm) affect the electrokinetic potential value so greatly that it is negative in the whole pH range.

The zeta potential decrease due to the *S. meliloti* 1021 EPS adsorption is primarily related to the slipping plane shift by loops and tails of the adsorbed polymer macromolecules. These structures are arranged perpendicularly to the adsorbent surface and contain many dissociated carboxylic groups, which will further affect the electrokinetic potential reduction. With the increase in the bacterial polysaccharide concentration, the decrease in the chromium(III) oxide particles zeta potential occurs. It is connected with the polymer chain conformation on the adsorbent surface. At higher EPS concentrations, polymer chains of the highest molecular weight adsorb on the Cr_2_O_3_ surface. Their macromolecules form loops and tails of greater lengths than the lower molecular weight macromolecules. This results in a stronger slipping plane shift, and thus lower ξ potential values. No difference in the Cr_2_O_3_ particles electrokinetic potential values in the EPS presence with the concentration of 10 and 50 ppm is probably connected with the adsorption of maximally long polymer chains and maximum slipping plane shift in both cases. Thus, at the EPS concentration equal to or above 10 ppm, the tail and loop structures of the adsorbed macromolecules have the largest size.

### Stabilization/Destabilization Mechanism of Chromium(III) Oxide Suspension With and Without *S. meliloti* 1021 Exopolysaccharide

Stabilization and destabilization mechanism of chromium(III) oxide suspension in the absence and presence of *S. meliloti* 1021 EPS has been explained on the basis of the results from the following measurements: (1) adsorption amount, (2) electrokinetic potential, (3) stability and (4) potentiometric titration. Knowledge about this mechanism is needed to assess EPS as a substance that makes Cr_2_O_3_ removal from water and wastewater more efficient. Adsorption and electrokinetic properties as well as stability of the chromium(III) oxide suspension containing *S. meliloti* 1021 EPS have not been described in the literature yet.

The chromium(III) oxide suspension without EPS is characterized by the highest stability at pH 3. It is associated with formation of the barrier of chloride ions coming from the supporting electrolyte solution (NaCl) around each positively charged metal oxide particle. This structure prevents particle collisions and thus the aggregate formation. In addition, the suspension stability is provided by the particles electrokinetic potential which is approximately +40 mV at pH 3. The above zeta potential value favours mutual repulsion of chromium(III) oxide particles and thus makes the suspension stable. This phenomenon is defined as electrostatic stabilization.

At pH 4.6, the chromium(III) oxide suspension is less stable than at pH 3. This is probably due to the lower value of the adsorbent electrokinetic potential (about +20 mV), so that the electrostatic repulsion forces between metal oxide particles are correspondingly weaker.

At pH 9 the system has stability close to pH 4.6. Under these conditions the barrier of sodium ions coming from the supporting electrolyte solution (NaCl) is formed around the solid particles having a negative charge. The Cr_2_O_3_ zeta potential value is approximately −22 mV.

On the other hand, at pH 7.6 the chromium(III) oxide suspension without microbial EPS is greatly unstable. This is certainly due to the adsorbent zero surface charge which is conducive to particle collision and formation of rapidly falling down aggregates.

Regardless of solution pH, *S. meliloti* 1021 EPS adsorption lowers the stability of the chromium(III) oxide suspension. At pH 3 and 4.6, destabilization occurs due to the metal oxide positive charge neutralization by the adsorbed polymer chains. As mentioned in the previous section, the EPS p*K*_a_ value is about 3.8, and its dissociation degrees at pH 3 and 4.6 are 0.14 and 0.86, respectively. Thus, at pH 3 the number of dissociated carboxylic groups is small, but sufficient to neutralize the particle surface charge. COO^−^ groups interact with all positive groups on the adsorbent surface providing complete solid coverage by polymer and its maximum adsorption. At pH 4.6 the number of dissociated functional groups is higher, making the chromium(III) oxide surface charge neutralization more likely. Changes in the electrokinetic potential values are indicative of destabilization mechanism. A 1 ppm EPS adsorption shifts the Cr_2_O_3_ isoelectric point approximately to 3.5. This probably means that at pH 3 and 4.6 the particles cease to repel, which allows the sedimenting flocs formation.

At pH 7.6 and 9 the suspensions have the lowest stability of all systems under examination. In both cases practically all carboxylic groups in the polysaccharide macromolecules are dissociated. However, the metal oxide surface charge is zero at pH 7.6 and negative at pH 9 which make the system destabilization by adsorbent charge neutralization impossible. At pH 7.6 and 9 suspension destabilization is the result of polymeric bridge formation by the adsorbed EPS macromolecules. Due to the EPS high molecular weight (HMW EPS) polymer chains have a length sufficient for their simultaneous adsorption on at least two solid particles. This definitely favours formation of flocs of significant sizes, which quickly fall to the bottom of the measuring vial. Such system destabilization by polymer bridging is defined as the bridging flocculation. It is certainly desirable in procedures for water purification and wastewater treatment.

**Fig. 8:**
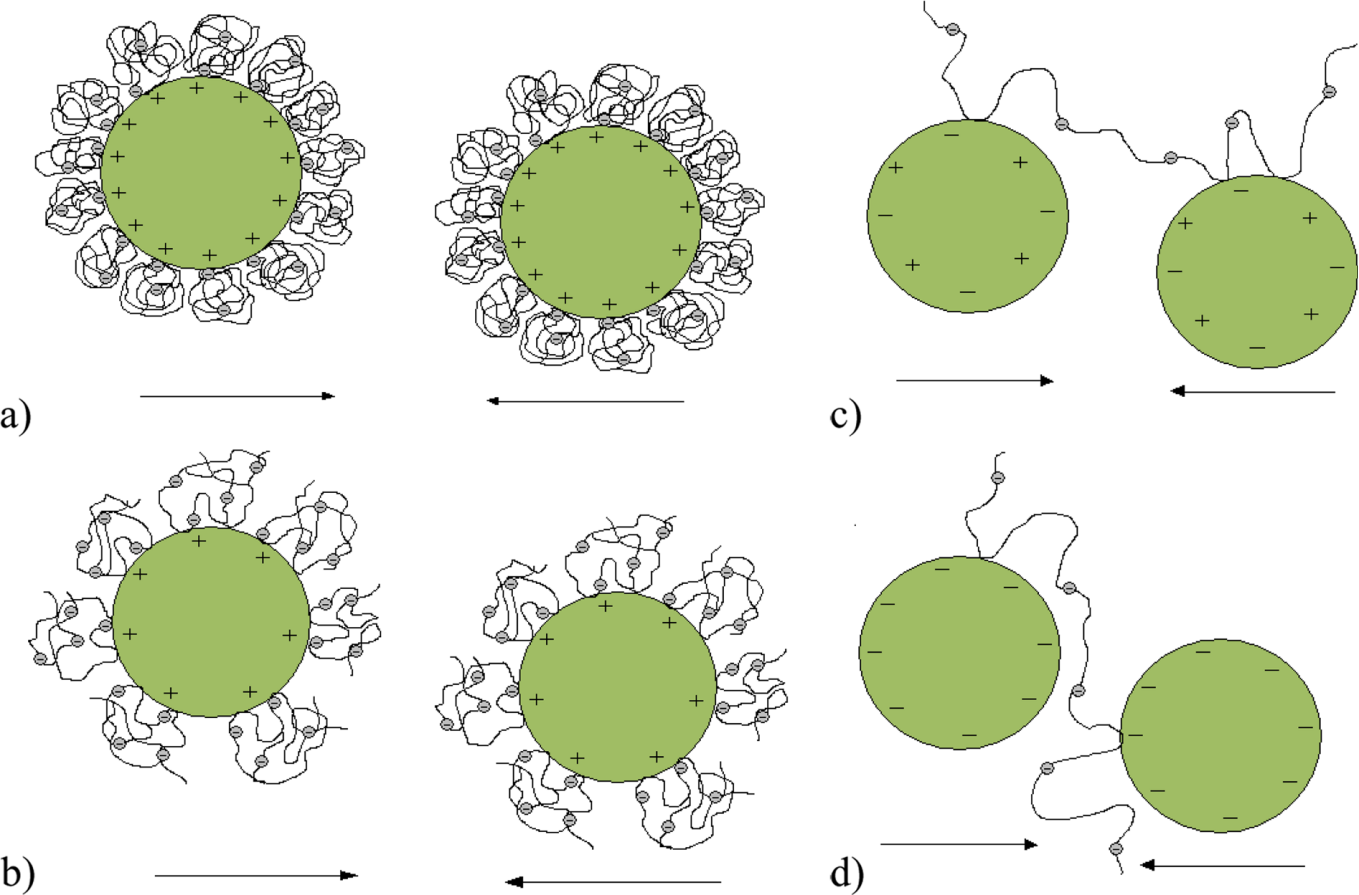
Destabilization mechanism of the Cr_2_O_3_ suspension by *Sinorhizobium meliloti* 1021 exopolysaccharide at pH: **a** 3, **b** 4.6, **c** 7.6, **d** 9. At pH 3 and 4.6 system destabilization is based on adsorbent charge neutralization by adsorbed macromolecules. At pH 7.6 and 9 there is bridging flocculation in the examined systems

The proposed destabilization mechanisms of the chromium(III) oxide suspension in the presence of *S. meliloti* 1021 exopolysaccharide in four examined solution pH values are shown schematically in Fig. 8.

## Conclusions

In order to determine the flocculation properties of *S. meliloti* 1021 EPS and the conditions that would allow for more efficient removal of chromium(III) oxide from sewages and wastewaters using EPS, adsorption, zeta potential and stability measurements as well as potentiometric titration were performed. As follows from the analysis of results: (1) the EPS p*K*_a_ is about 3.8, (2) the greatest EPS amount is adsorbed on the chromium(III) oxide surface at pH 3 and it decreases with the increasing pH, (3) EPS adsorption causes the Cr_2_O_3_ pH_pzc_ and pH_iep_ values shift to the acidic pH values, (4) the chromium(III) oxide suspension without the polymer is the most unstable at pH 7.6, i.e. under adsorbent zero surface charge conditions, (5) the Cr_2_O_3_ suspension with EPS is the most unstable at pH 7.6 and 9, which can be explained by bridging flocculation, (6) the biggest difference in the stability of the chromium(III) oxide suspension and aggregate (floc) sizes before and after the EPS addition was observed at pH 9.
